# A Replication Study Confirms the Association of Dendritic Cell Immunoreceptor (DCIR) Polymorphisms with ACPA - Negative RA in a Large Asian Cohort

**DOI:** 10.1371/journal.pone.0041228

**Published:** 2012-07-19

**Authors:** Jianping Guo, Xinyu Wu, Chun Lai Too, Fangrui Yin, Xiaolan Lu, Jing He, Ru Li, Xu Liu, Shahnaz Murad, Leonid Padyukov, Zhanguo Li

**Affiliations:** 1 Department of Rheumatology and Immunology, People's Hospital, Peking University, Beijing, China; 2 Rheumatology Unit, Department of Medicine, Karolinska Institutet, Stockholm, Sweden; 3 Institute for Medical Research, Kuala Lumpur, Malaysia; 4 Department of Rheumatology, the First Affiliated Hospital, Baotou Medical College, Baotou, China; 5 Department of Rheumatology, Xuanwu Hospital, Beijing, China; University of Southern California, United States of America

## Abstract

**Objectives:**

Dendritic cell immunoreceptor (*DCIR*) has been implicated in development of autoimmune disorders in rodent and *DCIR* polymorphisms were associated with anti-citrullinated proteins antibodies (ACPA)-negative rheumatoid arthritis (RA) in Swedish Caucasians. This study was undertaken to further investigate whether *DCIR* polymorphisms are also risk factors for the development of RA in four Asian populations originated from China and Malaysia.

**Methods:**

We genotyped two *DCIR* SNPs rs2377422 and rs10840759 in Han Chinese population (1,193 cases, 1,278 controls), to assess their association with RA. Subsequently, rs2377422 was further genotyped in three independent cohorts of Malaysian-Chinese subjects (MY_Chinese, 254 cases, 206 controls), Malay subjects (MY_ Malay, 515 cases, 986 controls), and Malaysian-Indian subjects (MY_Indian, 378 cases, 285 controls), to seek confirmation of association in various ethnic groups. Meta-analysis was preformed to evaluate the contribution of rs2377422 polymorphisms to the development of ACPA-negative RA in distinct ethnic groups. Finally, we carried out association analysis of rs2377422 polymorphisms with DCIR mRNA expression levels.

**Results:**

*DCIR* rs2377422 was found to be significantly associated with ACPA -negative RA in Han Chinese (OR 1.92, 95% CI 1.27–2.90, *P* = 0.0020). Meta-analysis confirms *DCIR* rs2377422 as a risk factor for ACPA-negative RA across distinct ethnic groups (OR_overall_ = 1.17, 95% CI 1.06–1.30, *P* = 0.003). The SNP rs2377422 polymorphism showed significant association with *DCIR* mRNA expression level, i.e. RA-risk CC genotype exhibit a significant increase in the expression of DCIR (*P* = 0.0023, Kruskal–Wallis).

**Conclusions:**

Our data provide evidence for association between *DCIR* rs2377422 and RA in non-Caucasian populations and confirm the influence of *DCIR* polymorphisms on RA susceptibility, especially on ACPA-negative RA.

## Introduction

Rheumatoid arthritis (RA) is a common autoimmune disease, characterized by chronic inflammation and progressive destruction in the joints. Although the pathogenesis of RA remains poorly understood, it is widely accepted that genetic risk factors contribute significantly to RA development. To date, over 30 RA susceptibility loci have been identified [1] and the most important genetic factor for RA was found in a group of the human leukocyte antigen (*HLA*)-*DRB1* alleles named as shared epitope (SE) [2]. Notably, the majority of RA susceptibility loci have been described as risk factors for anti-citrullinated protein antibodies (ACPA)-positive RA [2], [3], [4], [5], [6]. Direct comparison between disease subgroups revealed that different genetic association patterns existed between ACPA-positive and ACPA-negative RA, and little is known about the genetic contribution to ACPA-negative RA [7]. Moreover, recent discovered genetic loci for RA in one population were not always replicated in other ethnic groups, especially between European Caucasians and Asians [8], [9]. Thus, expanding the genetic study population(s) is needed to validate the existing genetic risk factors, and to understand the implication of genetic heterogeneity among the populations in RA.

The dendritic cell immunoreceptor (*DCIR*) gene, located on human chromosome 12p13, is a member of the C-type lectin family and structurally characterized by a carbohydrate recognition domain (CRD) and signaling through an immunoreceptor tyrosine-based inhibitory motif (ITIM) [10]. Its precise role and function are not completely understood, but we and others have previously identified a cluster of C-type lectin receptor genes including *DCIR* that regulates arthritis susceptibility and influences the development of infectious diseases in rat [11], [12]. DCIR knockout (DCIR-KO) mice showed a markedly exacerbated response to collagen-induced arthritis, and aged DCIR-KO mice spontaneously developed sialadenitis and enthesitis with elevated levels of autoantibodies [13]. In human, four *DCIR* single nucleotide polymorphisms (SNPs) rs2024301, rs2377422, rs1133104, and rs10840759 which located in 3 different recombination blocks, were significantly associated with RA susceptibility, in ACPA-negative RA subset in the Swedish population [14]. However, this locus did not reach the genome-wide significant level in recently performed GWAS for ACPA-negative RA [7]. It also remains unclear whether this ACPA-negative RA association is valid in other ethnic groups, especially in non-Caucasians. On this basis, the aim of this study was to investigate the possible association of *DCIR* polymorphisms with ACPA-positive and ACPA-negative RA in four independent Asian populations originated from China and Malaysia.

## Results

Both SNPs rs2377422 and rs10840759 were in HWE (*P*>0.05) in cases and controls of Han Chinese cohort, with the study power of 0.986 to detect the modest effect size with OR = 1.40, and a fixed minor allele frequency of 40% for RA association. In MyEIRA study, rs2377422 was in HWE (*P*>0.05) in all three cohorts, except for the controls in MY_Malay (*P* = 0.0024). Taking together, the MyEIRA study has a statistical power of 0.989 to detect the significant effect between rs2377422 and RA. However, the single-population study power was generally low, except for the Malay population (study power = 0.867).

### Association of *DCIR* SNP rs2377422 with RA in multiple Asian ethnic groups

We first sought to replicate SNPs rs2377422 and rs10840759 in Han Chinese cohort. The distribution of both allele and genotype frequencies was shown in Table 1.While the previously reported RA risk SNP rs10840759 showed no association with RA in our cohort (allele model: *P* = 0.93, OR 0.99; 95% CI 0.89–1.12; genotype model: *P* = 0.68, OR 1.05; 95% CI 0.83–1.32), significant association with RA was observed for SNP rs2377422, both at allele model (risk allele C, *P* = 7.4×10^−3^, OR 1.17; 95%CI 1.04–1.31) and at genotype model (recessive model CC vs. TT+TC, *P* = 9.0×10^−3^, OR 1.37; 95%CI 1.08–1.73). Genotyping of rs2377422 across three ethnic populations from Malaysia however, revealed no association between rs2377422 and RA susceptibility, in the three sample sets separately (*P* = 0.29, OR 0.93; 95%CI 0.89–1.51, for MY_Chinese, *P* = 0.39; OR 0.93; 95%CI 0.79–1.09 for MY_Malay and *P* = 0.39, OR 1.02; 95% CI 0.93–1.12, for MY_Indian, respectively), or in the meta-analysis within the Malaysian cohorts (allele model: OR_overall_ = 0.98, 95% CI 0.88–1.10; *P_het_* = 0.38, *I^2^* = 3%, data not shown).

**Table 1:** Association analysis of rs2377422 with RA, adjusting for sex and age.

		Control	RA	OR (95% CI)	*P* value
**Han Chinese**					
rs2377422		n = 1278	n = 1193		
allelic	T/C	1386/1170	1202/ 1184	1.17 (1.04–1.31)	0.0074
genotypic	TT+TC/CC	368+650/260	309+584/ 300	1.37 (1.08–1.73)	0.0090
rs10840759		n = 1276	n = 1128		
allelic	T/C	1265/1287	1121/ 1135	0.99 (0.89–1.12)	0.93
genotypic	TT+TC/CC	315+657/304	299+537/ 292	1.05 (0.83–1.32)	0.68
**MY_ Chinese**					
rs2377422		n = 202	n = 250		
allelic	T/C	174/230	234/ 266	0.86 (0.66–1.12)	0.26
genotypic	TT+TC/CC	36+102/64	56+122/ 72	1.15 (0.77–1.72)	0.58
**MY_ Malay**					
rs2377422		n = 973	n = 510		
allelic	T/C	675/1271	337/ 683	1.07 (0.92–1.26)	0.37
genotypic	TT+TC/CC	139+397/437	59+219/ 232	0.98 (0.79–1.21)	0.88
**MY_ Indian**					
rs2377422		n = 282	n = 373		
allelic	T/C	227/337	309/ 437	0.95 (0.76–1.19)	0.67
genotypic	TT+TC/CC	45+137/100	58+193/ 122	1.13 (0.82–1.57)	0.51

RA: rheumatoid arthritis; OR (95% CI): odds ratio (95% confidence interval); MY_Chinese: Malaysian Chinese; MY_Malay: Malays; MY_Indian: Malaysian Indian.

### SNP rs2377422 polymorphism conferred a higher risk for developing ACPA-negative RA

Following stratification for ACPA status, we found a significant association of rs2377422 with ACPA-negative RA in Han Chinese (CC vs. TT+TC: OR 1.92, 95% CI 1.27–2.90, *P* = 0.0020, Table 2), despite loss of power in the analysis. No association between rs2377422 and the subsets of RA was found in the three Malaysian sample sets when analyzed separately (shown in Table 2) or in the meta-analysis for ACPA-negative RA within these sample sets (allele model: OR_overall_ = 0.88, 95% CI 0.71–1.10; *P_het_* = 0.94, *I^2^* = 0%, data not shown). A weak association of rs2377422 with ACPA-positive RA was also observed in Han Chinese cohort, but did not reach statistical significance (OR 1.34, 95% CI 0.99–1.82, *P* = 0.058).

**Table 2:** RA association of rs2377422 according to ACPA status, adjusting for sex and age.

Groups	rs2377422		Adjusted OR (95% CI)	Adjusted *P*
	TT+TC	CC		
**Han Chinese**				
Controls	368+650	260	referent	
ACPA++	133+227	109	1.34 (0.99–1.82)	0.058
ACPA−	36+81	58	1.92 (1.27–2.90)	0.0020
**MY_ Chinese**				
Controls	36+102	64	referent	
ACPA+	39+78	49	0.90 (0.58–1.41)	0.60
ACPA−	17+44	23	0.81 (0.46–1.43)	0.47
**MY_ Malay**				
Controls	139+397	437	referent	
ACPA+	37+130	140	1.03 (0.79–1.33)	0.83
ACPA−	22+89	92	1.02 (0.75–1.38)	0.92
**MY_ Indian**				
Controls	45+137	100	referent	
ACPA+	38+126	86	0.95 (0.67–1.36)	0.80
ACPA−	20+67	36	0.75 (0.22–1.50)	0.23

RA: rheumatoid arthritis; OR (95% CI): odds ratio (95% confidence interval); MY_Chinese: Malaysian Chinese; MY_Malay: Malays; MY_Indian: Malaysian Indian; ACPA: anti-citrullinated proteins antibodies; +/−: positive/negative.

### Meta-analysis confirmed *DCIR* rs2377422 as a risk factor for ACPA-negative RA across multiple ethnic groups

**Figure 1:**
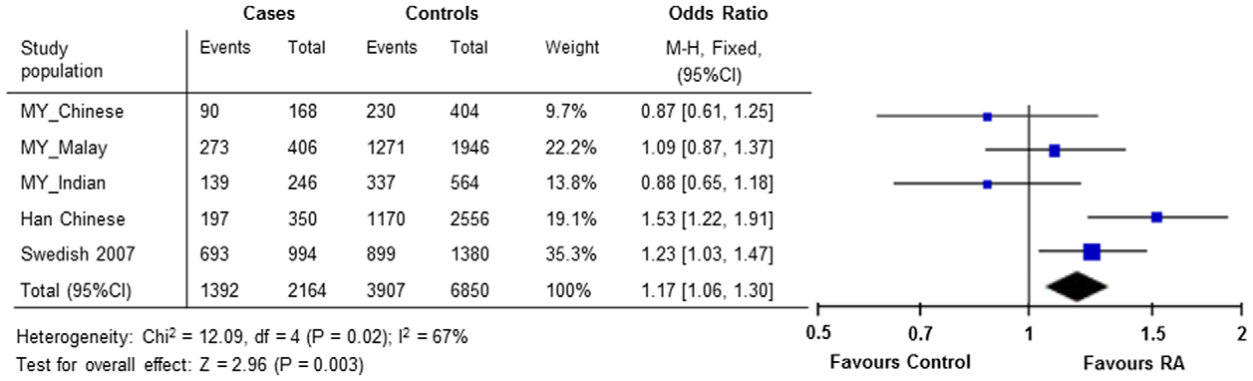
Meta-analysis of rs2377422 for ACPA-negative RA across different ethnic groups. Combined OR values from Han Chinese, Malaysian Chinese, Malays, Malaysian Indian and Swedish rheumatoid arthritis (RA) case–control cohorts.

For a better estimation of rs2377422 polymorphisms contributed to the development of ACPA-negative RA, we preformed a meta-analysis considered the current RA datasets, as well as the data reported by Lorentzen, *et al.* [14]. As shown in Figure 1, the allele model meta-analysis for rs2377422 showed a significant association with ACPA-negative RA under a fixed effects model (OR_overall_ = 1.17, 95% CI 1.06–1.30, *P* = 0.003) but not in random effects model (OR_overall_ = 1.13, 95% CI 0.93–1.36, *P* = 0.23, data not shown), due to a significant heterogeneity observed across different ethnic groups (*P_het_* = 0.02, *I^2^* = 67%).

### DCIR transcription quantification and its association with rs2377422 genotypes

**Figure 2:**
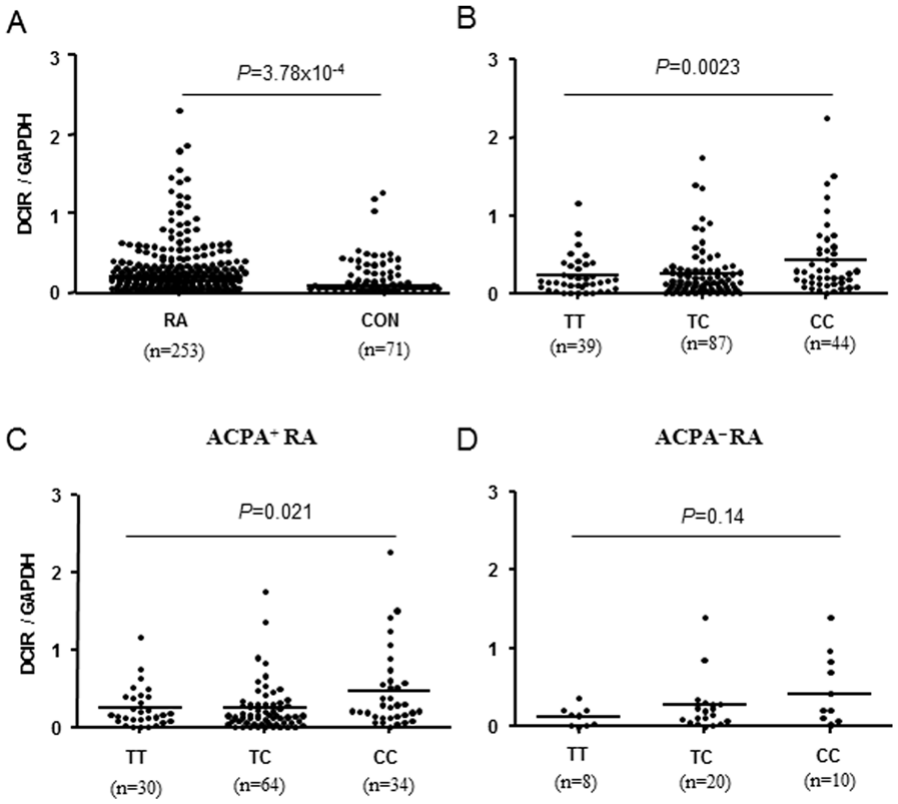
The *DCIR* mRNA expression in peripheral blood mononuclear cells (PBMCs) from patients with rheumatoid arthritis (RA) according to rs2377422 genotype. (A) Expression of *DCIR* mRNA was assessed by quantitative real-time PCR in freshly isolated PBMCs. *DCIR* mRNA level was significantly elevated in RA cases, compared with healthy controls (Mann–Whitney U test, *P* = 3.78×10^−4^). (B) TC or CC genotype of SNP rs2377422 had significantly higher levels of DCIR expression, compared with data from genotype TT (*P* = 0.0032, Kruskal–Wallis). Following stratification for ACPA status, a similar association pattern was observed for both ACPA-positive (*P* = 0.021, Kruskal–Wallis) (C) and ACPA-negative RA (*P* = 0.14, Kruskal–Wallis) (D).

First, we performed the analyses of *DCIR* mRNA expression in RA cases and in healthy controls. As shown in Figure 2A, *DCIR* expression level was significantly elevated in RA cases, compared with healthy controls (0.47±0.10 vs. 0.17±0.03, *P* = 3.78×10^−4^, Mann–Whitney U test). To study the effect of rs2377422 variation on *DCIR* gene expression, taking into account on ACPA status, *DCIR* mRNA levels were analyzed for RA cases with different genotypes at inclusion. As shown in Figure 2B, the individuals with the TC or CC genotype of SNP rs2377422 had significantly higher levels of *DCIR* expression, compared with data from genotype TT (*P* = 0.0023, Kruskal–Wallis). Following stratification for ACPA status, a similar association pattern was also observed in both ACPA-positive (*P* = 0.021, Kruskal–Wallis) and ACPA-negative RA (*P* = 0.14, Kruskal–Wallis), though the latter did not reach the statistic significance due to the loss of power in the analysis.

In addition, we analyzed the association between *DCIR* expression level and several common RA phenotypes in patients. However, in our material, we did not find a correlation between *DCIR* expression and disease duration (n = 233, r = 0.128, *P* = 0.107), or disease activity (DAS28) (n = 197, r = 0.067, *P* = 0.503). Also, no correlation was observed between *DCIR* expression and the level of anti-CCP antibody (n = 166, r = 0.046, *P* = 0.616) (data not shown).

## Discussion

The *DCIR* SNP rs2377422 was initially detected as a susceptibility factor for ACPA-negative RA in the Swedish population. With the aim of validating the initially reported association of *DCIR* polymorphisms with RA in the Caucasian population, we conducted a large case-control study involving 5,075 subjects from four independent Asian populations. In accordance with the report by Lorentzen *et al*, our study supports the implication of *DCIR* SNP rs2377422 in RA susceptibility. Stratification by ACPA status confirmed the significant association of *DCIR* rs2377422 with ACPA-negative RA in Han Chinese population. Our meta-analysis provides further confirmation for an association between rs2377422 and ACPA-negative RA. Importantly, our study demonstrates that rs2377422 may affect *DCIR* expression in primary mononuclear cells, with potential implications for the disease pathogenesis.

While there is a significant association between *DCIR* polymorphisms and ACPA-negative RA in the Han Chinese sample set in this study and that previously reported in Swedish population, there was insignificant risk of rs2377422 regarding ACPA-negative RA among the three major ethnic groups from Malaysia. The reason for this heterogeneous effect is unclear. It is more likely due to genetic and/or environmental and/or clinical heterogeneity between populations and/or inadequate power of the individual sample sets [15]. A recent study has reported that even Han Chinese population is complicatedly substructured, with the main observed clusters corresponding roughly to northern Han and southern Han. Simulated case-control studies showed that the genetic differentiation among these clusters is sufficient to lead to an inflated rate of false-positive results [16]. Interestingly, in present study, all the Han Chinese cases and controls were originated from northern China (Beijing, Hebei, Liaoning and Heilongjiang provinces), whereas the Chinese cases and controls from MyEIRA study are mainly the descendents from southern China (Fujian, Guangdong and Hainan provinces). It may, at least partly, explain the inconsistent results in these two Chinese cohorts. It also indicates that association of rs2377422 with RA should be generalized to additional populations with caution.

The previously detected association of *DCIR* SNP rs10840759 with ACPA-negative RA in subjects of European ancestry was not found in Han Chinese population. One possible explanation is that this SNP has a different LD structure within *DCIR* in different ethnic groups. Based on dbSNP data (build 129), rs10840759 has stronger LD with rs2377422 in the population of European ancestry (D′ = 0.68, r^2^ = 0.30) than that in Han Chinese population (D′ = 0.38, r^2^ = 0.10). The other initially reported RA-associated SNP, rs1133104 also has stronger LD with SNP rs2377422 in the population of European ancestry (D′ = 0.74, r^2^ = 0.34) than that in Han Chinese population (D′ = 0.35, r^2^ = 0). In this work, rs1133104 and another candidate risk SNP rs2024301 were not genotyped, due to the low frequency or monomorphic in Han Chinese population.

The SNP rs2377422 resides in the second intron of the *DCIR* gene and its functional consequence remains to be elucidated. Given that abundant *DCIR* expression was observed in rheumatic joints and decreased *DCIR* expression was accompanied by disease amelioration [17]. As a first attempt to study this issue, we looked at the pattern of *DCIR* mRNA expression between RA patients and healthy controls and a possible association between rs2377422 polymorphism and *DCIR* expression in PBMCs. In our cohort, we observed *DCIR* expression level was significantly elevated in RA cases, compared with healthy controls. In RA cases, individuals with TC or CC genotype had significantly increased mRNA levels compared with those with the TT genotype. This result might be biologically plausible since the allele C association with increased *DCIR* expression was found to be overrepresented in RA cases in both Han Chinese and Swedish populations. Interestingly, Ronninger *et al.* reported that a comparison of mRNA expression levels in different *DCIR* transcripts revealed no significant difference between RA cases and controls in their cohort [18]. The disconcordance may be due to the different criteria for case selection and/or different sample sizes. In current expression cohort, all cases were active RA (DAS28 = 4.7±0.7, n = 253). Furthermore, in the presence of allele C, the *DCIR* expression was higher in both ACPA-positive and ACPA-negative RA subsets, though being unable to reach the statistical significance in ACPA-negative RA due to the loss of statistical power, indicating rs2377422 might interact with other risk factor(s) to contribute specifically to ACPA-negative RA, as the contribution of *PTPN22* to ACPA-positive RA [4]. Moreover, in Eklöw's study, almost all cell types were shown to express DCIR protein [17], but *DCIR* mRNA expression was not previously systematically investigated in subpopulations of human cells. Theoretically, it is possible that the profile of the expression could be changed by a single type of cells with very high expression of DCIR. It should be further investigated, but from the known protein expression data it looks unlikely.

One of the limitations of the current study is that we did not comprehensively investigate *DCIR* polymorphisms with sufficient density. Given that there may be multiple risk polymorphisms within one gene, other *DCIR* genetic variants might also contribute to the development of RA. The ACPA-negative RA associated intronic SNP rs2377422 is unlikely to be the causal variant and rather is more likely to be in strong LD with the biologically relevant variant. Further fine-mapping and more functional studies are needed to elucidate the association.

In conclusion, our data confirm the influence of *DCIR* polymorphisms in RA susceptibility, especially in the subset of patients negative for ACPA.

## Materials and Methods

### Study subjects

The baseline demographic characteristics of patients and controls are shown in Table 3. In Han Chinese cohort, a total of 1,193 patients with RA (mean age 52.3±13.2 years; 78.1% females), were recruited from the Department of Rheumatology at Peking University People's Hospital, Xuanwu Hospital and Peking University Third Hospital. All patients satisfied the American College of Rheumatology 1987 revised criteria for a diagnosis of RA [19]. In which, 72.8% (469/644) were ACPA-positive, defined and quantified using a second generation anti-CCP (anti-cyclic citrullinated peptides) antibodies ELISA kit (Euroimmun, Luebeck, Germany). Samples with results >5 RU/mL were defined as positive. The control group comprised 1,278 unrelated healthy individuals (mean age 42.1±9.4 years; 69.8% females) and was recruited from Health Care Centers from Peking University People's Hospital. All patients and healthy controls were Han Chinese originated from northern China.

**Table 3:** Demographic characteristic of the study cohorts.

Characteristic	Han Chinese	MY_ Chinese	My_Malay	MY_ Indian
Origin	China	Malaysia	Malaysia	Malaysia
No. of patients	1193	254	515	378
No. of controls	1278	206	986	285
Female sex (patients, %)	78.1	83.3	85.7	87.5
Female sex (controls, %)	69.8	87.5	88.9	85.7
Age of patients (mean ± SD yrs)	52.4±13.2	52.6±11.2	46.2±11.8	47.7±10.9
Age of controls	42.1±9.4	50.9±11.5	46.3±11.3	48.1±10.6
Disease duration (mean ± SD yrs)	7.5±7.2	1.2±1.9	1.0±2.0	1.1±1.5
ACPA-positive (%)	72.8	66.4	60.2	67.0

MY_Chinese: Malaysian Chinese; MY_Malay: Malays; MY_Indian: Malaysian Indian; SD: standard deviation; ACPA: anti-citrullinated proteins antibodies.

The Malaysian cohort was derived from the Malaysian Epidemiological Investigation of Rheumatoid Arthritis (MyEIRA) case-control study. The details of MyEIRA study was described elsewhere [20]. In brief, 1,147 early RA cases and 1,477 sex, age and residential area matched controls were included. All RA cases were diagnosed by rheumatologists and fulfilled the 1987 ACR criteria. The median disease duration for RA was one year (inter quartile range = 2 years). The MyEIRA study comprising of three major ethnic groups of Asian descent: Malays, Chinese and Indian. The distribution of these different ethnic groups is presented in Table 3. Data for ACPA status were available for all RA cases and controls in the MyEIRA study.

The study was approved by the local ethical committees at each institution (Peking University People's Hospital and the Ministry of Health, Malaysia, respectively), and written informed consents were obtained from all participants.

### SNP selection and genotyping

Within the DCIR gene, SNPs with allele frequencies (MAF>0.05) in HapMap CHB are all located in introns, except for one nonsynonymous SNP: rs2024301 and one SNP in 3′ UTR: rs10840759 (http://hapmap.ncbi.nlm.nih.gov). Given that RA candidate SNP rs2024301 is monomorphic and rs1133104 is rare variant in Han Chinese population, only the RA candidate risk SNPs rs2377422 and rs10840759 were selected for the present study.

The TaqMan Genotyping Assays were applied for genotyping of SNP rs2377422 (predesigned ID: C_16001297_10, Applied Biosystems, ABI) and rs10840759 (custom-designed ID: AHX0ZWL, Forward primer: 5′- CTCTTAATTTTTATCTGGTTGCTAAAGAATTATTTACCAA-3′;

Reverse primer: 5′-AGTATATATATACAATTATATATCAGTATAGTAGGGATGAAGAGAAAA -3′, ABI). The end point fluorescence readings were performed using an ABI Prism 7300 System. The genotyping successful rates of rs2377422 and rs10840759 were 98.2% and 97%, respectively.

### DCIR transcription quantification

A total of 253 patients with RA and 71 healthy individuals were assessed for DCIR mRNA expression in peripheral blood mononuclear cells (PBMCs). All cases and controls are Han Chinese. All cases were active RA (DAS28 = 4.7±0.7), and 170 of RA cases were derived from 1193 RA samples and had genotyping data for rs2377422.

Cells were harvested and total RNA was extracted using RNeasy mini Kit (Qiagen, Stanford, CA). Reverse transcription reactions were performed using RevertAid™ First Strand cDNA Synthesis Kit (Thermo Scientific Fermentas, Shenzhen, China). Primers were designed using the Primer Express software (Perkin-Elmer, Wellesley, MA) constructed over cDNA sequence (access from NCBI, Entrez Nucleotide) exon/exon boundaries to avoid amplification of contaminating genomic DNA, and ordered from SBS Genetech (Beijing, China). Housekeeping gene GAPDH was used as endogenous control.

Primers used were:

DCIR: Forward: 5′-GACCCTCACACTCAGATCATC-3′,

Reverse: 5′-ACGCTGGCTCAGCCACTC-3′.

GAPDH: Forward: 5′-TCAACTACATGGTCTACATGTTCCAG-3′,

Reverse: 5′-TCCCATTCTCAGCCTTGACTG-3′.

The human *DCIR* gene comprises of six exons, and at least, has four known transcripts (DCIR_v1, DCIR_v2, DCIR_v3 and DCIR_v4) (http://www.ncbi.nlm.nih.gov/gene). In present study, the primer pair for *DCIR* was designed in the first and the second exons targeting both DCIR_v1 and DCIR_v2 transcripts, taking account of the abundance of transcripts [18].

Samples were analyzed individually. Amplification and quantitative analyses were performed using ABI 7300 Real-Time PCR system, and SYBR green methodology (SYBR Green Supermix, ABI), respectively. Relative quantification of mRNA levels was calculated (7500 Sequence Detection System Software Version 1.4, ABI) using standard curves generated using five serial dilutions (i.e. 1∶1, 1∶5, 1∶25, 1∶125, 1∶625). Samples were run in duplicate. After computing relative amount of target and endogenous control, the final relative mRNA quantities of targets were represented as the ratio between the target and endogenous GAPDH.

### Statistical analyses

The Hardy-Weinberg equilibrium (HWE) test was performed for each polymorphism, using Pearson's goodness-of-fit chi-square test. The Pearson chi-square tests were performed for the comparisons of allelic frequency differences between cases and controls. The odds ratios (OR) and 95% confidence intervals (CI) for alternative genetic model (recessive model) analysis were calculated using logistic regression, adjusting for age and sex. The Mann-Whitney U test was applied for the analysis of the transcription levels between RA and control groups. Association of *DCIR* expression with genotypic variants was analyzed using Kruskal–Wallis test. All statistical analyses were conducted using program SPSS 13.0 (SPSS Inc., Chicago, IL, USA). The *P*<0.05 after Bonferroni correction was considered statistically significant.

### Power analysis

The power analyses were performed retrospectively for the available samples (cases and controls), using a fixed minor allele frequency of 40%, a Type I error *P* of 0.05, and an OR of 1.4. The PS software (version 3.0.14) was used for power calculation (available at http://www.mc.vanderbilt.edu/prevmed/ps).

### Meta-analysis

Meta-analysis was performed using Review Manager 5 software (www.cc-ims.net/RevMan) and carried out with the Mantel-Haenszel method. A significant *I* ^2^ statistic (*I* ^2^>30%, *P*<0.05) indicated heterogeneity for ORs across studies. The fixed-effects model was applied in current meta-analyses. The association data between SNP rs2377422 and ACPA-negative RA in Swedish population was cited from the publication by Lorentzen, *et al.* [14].
