# Correction: A Replication Study Confirms the Association of Dendritic Cell Immunoreceptor (DCIR) Polymorphisms with ACPA - Negative RA in a Large Asian Cohort

**DOI:** 10.1371/annotation/2ca25d9c-7347-4b09-bd7a-09d6d37ff322

**Published:** 2012-10-08

**Authors:** Jianping Guo, Xinyu Wu, Chun Lai Too, Fangrui Yin, Xiaolan Lu, Jing He, Ru Li, Xu Liu, Shahnaz Murad, Leonid Padyukov, Zhanguo Li

There was an error in the Funding section. The correction funding information is as follows: This study was financially supported by National Basic Research Program of China (973 Program) (No. 2010CB529105, http://www.973.gov.cn/AreaAppl.aspx), [^] Program of International Science & Technology Cooperation from MOST (No:2010DFB34000, http://www.most.gov.cn/), [^] Major International Joint Research Project from NSFC (No: 81120108020 http://www.nsfc.gov.cn/Portal0/default152.htm) [^] and Doctoral Fund of Ministry of Education of China (No: 20110001110045, http://www.cutech.edu.cn/cn/index.htm), [^] Beijing Natural Science Foundation (No:7122196, http://www.bjnsf.org/). [^] MyEIRA study was financially supported by the Ministry of Health (MOH), Malaysia: MRG 7/2005 and JPP-IMR 08-012(NMRR-08-820-1975), and by the Swedish Medical Research Council and the Swedish Combined program. The funders had no role in study design, data collection and analysis, decision to publish, or preparation of the manuscript.

Also, a portion of text from the Materials and Methods section is missing from the PDF version of this article, but is correct and present in the online version. The complete text of the sections "SNP selection and genotyping" and "DCIR transcription quantification" is:

**SNP selection and genotyping**

Within the DCIR gene, SNPs with allele frequencies (MAF>0.05) in HapMap CHB are all located in introns, except for one nonsynonymous SNP: rs2024301 and one SNP in 3′ UTR: rs10840759 (http://hapmap.ncbi.nlm.nih.gov). [^] Given that RA candidate SNP rs2024301 is monomorphic and rs1133104 is rare variant in Han Chinese population, only the RA candidate risk SNPs rs2377422 and rs10840759 were selected for the present study.

The TaqMan Genotyping Assays were applied for genotyping of SNP rs2377422 (predesigned ID: C_16001297_10, Applied Biosystems, ABI) and rs10840759 (custom-designed ID: AHX0ZWL, Forward primer: 5′- CTCTTAATTTTTATCTGGTTGCTAAAGAATTATTTACCAA​-3′;

Reverse primer: 5′-AGTATATATATACAATTATATATCAGTATAGTAGGGA​TGAAGAGAAAA-3′, ABI). The end point fluorescence readings were performed using an ABI Prism 7300 System. The genotyping successful rates of rs2377422 and rs10840759 were 98.2% and 97%, respectively.

**DCIR transcription quantification**

A total of 253 patients with RA and 71 healthy individuals were assessed for DCIR mRNA expression in peripheral blood mononuclear cells (PBMCs). All cases and controls are Han Chinese. All cases were active RA (DAS28 = 4.7±0.7), and 170 of RA cases were derived from 1193 RA samples and had genotyping data for rs2377422.

Cells were harvested and total RNA was extracted using RNeasy mini Kit (Qiagen, Stanford, CA). Reverse transcription reactions were performed using RevertAid™ First Strand cDNA Synthesis Kit (Thermo Scientific Fermentas, Shenzhen, China). Primers were designed using the Primer Express software (Perkin-Elmer, Wellesley, MA) constructed over cDNA sequence (access from NCBI, Entrez Nucleotide) exon/exon boundaries to avoid amplification of contaminating genomic DNA, and ordered from SBS Genetech (Beijing, China). Housekeeping gene GAPDH was used as endogenous control.

Primers used were:

DCIR: Forward: 5′-GACCCTCACACTCAGATCATC-3′,

Reverse: 5′-ACGCTGGCTCAGCCACTC-3′.

GAPDH: Forward: 5′-TCAACTACATGGTCTACATGTTCCAG-3′,

Reverse: 5′-TCCCATTCTCAGCCTTGACTG-3′.

The human DCIR gene comprises of six exons, and at least, has four known transcripts (DCIR_v1, DCIR_v2, DCIR_v3 and DCIR_v4) (http://www.ncbi.nlm.nih.gov/gene). [^] In present study, the primer pair for DCIR was designed in the first and the second exons targeting both DCIR_v1 and DCIR_v2 transcripts, taking account of the abundance of transcripts [18].

Samples were analyzed individually. Amplification and quantitative analyses were performed using ABI 7300 Real-Time PCR system, and SYBR green methodology (SYBR Green Supermix, ABI), respectively. Relative quantification of mRNA levels was calculated (7500 Sequence Detection System Software Version 1.4, ABI) using standard curves generated using five serial dilutions (i.e. 1:1, 1:5, 1:25, 1:125, 1:625). Samples were run in duplicate. After computing relative amount of target and endogenous control, the final relative mRNA quantities of targets were represented as the ratio between the target and endogenous GAPDH. 

